# The COVID-19 containment in Vietnam: What are we doing?

**DOI:** 10.7189/jogh.10.010338

**Published:** 2020-06

**Authors:** Toan Luu Duc Huynh

**Affiliations:** School of Banking, University of Economics Ho Chi Minh City, Vietnam

This viewpoint provides an explanation from the public health policies of Vietnamese government to contain the contagious disease with regard to COVID-19 pandemic. A combination of early lockdown, increase in the “virality” of health information, encouragement in health declaration, regulation for wearing mask in the public, and country’s unity have been the effective ways to cope with this deadly virus in Vietnam, a developing country, which became the first country to halt the SARS spread successfully in 2003.

Obviously, many people will ask the question of why we should care about Vietnam in the fighting campaign against COVID-19. Before answering this question, I will stress that an outbreak of severe acute respiratory syndrome (SARS), a fatal infectious disease, became a global emergency in 2003. At that time, Vietnam became the first country to halt the SARS spread successfully. Thus, Vietnam is of interest to the epidemic health policies as a case study of successful epidemic containment. The COVID-19 pandemic was confirmed to have spread within Vietnam on January 23, 2020. At the time of writing this article, there were no confirmed deaths in this country. From January 23, 2020 to February 13, 2020 (ie, 21 days), the country only recorded 16 confirmed cases. In the next 22 days, there were no new cases in Vietnam. Although the numbers of confirmed cases are now increasing daily, the rate of the increase remains below 15% since the first case was detected. The Vietnamese government declared that COVID-19 is initially under the control. In addition, the risk perception of COVID-19 among Vietnamese citizens is higher than the average [1]. This viewpoint will discuss potential reasons for these findings, which might be helpful for many countries to implement as the appropriate policies. Especially, the director of World Health Organization, Tedros Adhanom, appreciated the timely policies and regulations which were implemented in Vietnam.

**Figure 1** summarizes the timely policies to cope with the spread of the COVID-19 pandemic. When the first case was found in Vietnam, the Vietnamese government chose to close the border with China by canceling all flights from Wuhan. Besides, the following actions were made based on the global situation, shown in **Figure 1**. In summary, the government had considered the COVID-19 pandemic as a serious concern from the beginning. They primed the Vietnamese citizens to become more cautious about the worst-case scenario of the major public health crisis. This is a clear lesson for many European countries, which overlook the COVID-19 at the first stage. Therefore, Vietnam did well to use ‘*the golden time*’ to contain the spread of the novel coronavirus. While the recent study by La et al. [2] emphasized the role of official news from the newspaper, socioeconomic aspects, and academic research, this viewpoint will offer more insights about the “nudging behaviors”, which are widely applied in behavioral and health economics to call people to do things right.

**Figure 1:**
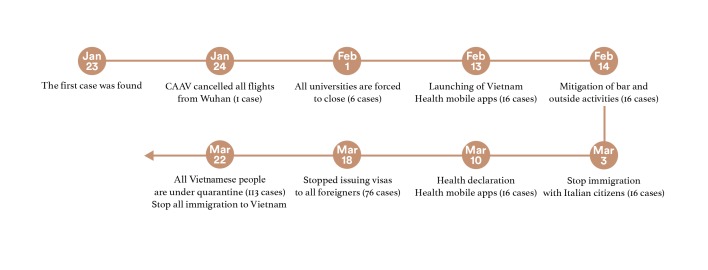
The summary timeline of nationally implemented policies in Vietnam. CAAV – Civil Aviation Administration of Vietnam.

**Figure:**
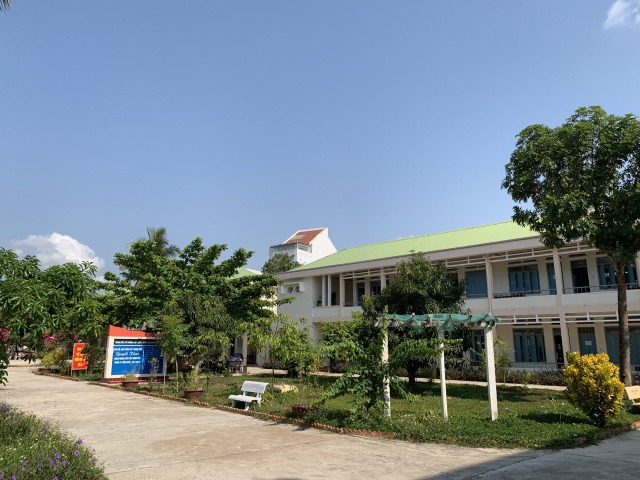
Photo: The compulsory quarantine area for Vietnamese citizens who came back from overseas (from the author’s own collection, used with permission).

Instead of causing the mass panic, the Vietnam authorities launched the COVID-19 video going viral as a prevention message. The song, a collaboration between Vietnam’s Health Ministry and musician, has been viewed more than thirty-two million times on YouTube [3]. The song evokes healthy practices to wash the hands and not to touch the face regularly. After that, the song went “viral” and was broadcasted on American television and on many European channels. This was also a part of the national campaign to deliver health policy messages to people. Instead of forcing the citizens with the compulsory regulation, the Vietnam government did well to disseminate nudging-behavior policies, which can increase the “virality” of the information [4].

Also, the Vietnamese authorities continuously informed the citizens through many methods, such as mobile phone messages. According to the Ministry of Information and Technology, about 6 billion messages have been sent to the Vietnamese citizens to raise their awareness about the hand-washing, self-quarantine, self-checking health, etc. Notably, on March 19, 2020, there were 1 040 000 downloads of the mobile application “NCOVI”, then 146.741 health declaration forms (paper-pencils), and 378 000 online declarations via Vietnam Health Declaration application. It meant that the use of modern technology in the fight against the COVID-19 in Vietnam was well executed.

Moreover, this viewpoint will also briefly discuss the role of wearing medical masks in Vietnam. The recent study of Huynh [5] found that Vietnamese people wear mask because of the high risk perception. On March 16, 2020, the Vietnamese government requested that everyone should wear face masks when going into public spaces to protect themselves and others. It was not only an encouraging message, but also the regulation applied to every citizen. Although World Health Organization recommended that only positively-confirmed patients should wear medical masks to mitigate COVID-19 transmission, it seems that in several Asian countries mask adoption seems to correlate with slowing down the pace of COVID-19 transmission. China, South Korea and Vietnam are all good examples.

Finally, the campaign of fighting the COVID-19 is not the sole duty of the Vietnamese government. This is the first time since the unification after the Vietnam War that the whole Vietnamese community, including military, scholars, businessmen, and many different classes in our society, have participated in different types of contributions. There are many universities and student dormitories which are used as the quarantine areas. The soldiers are encouraged to become public servants to supervise those who come back from the epidemic areas in quarantine areas. The businessmen are raising the movement to donate lands and provide financial incentives to fight against the COVID-19 outbreaks. Our last point means that Vietnam does not look at COVID-19 as the challenge in violation of our citizens’ freedom. What we are learning from the event is the importance of our country's unity. Therefore, according to the international survey conducted by the 12 different institutions, including Harvard, Cambridge, IESE, and Warwick University, Vietnam is ranked the second position that participants trust their country’s government to take care of its citizens [6].

## CONCLUSION

Although the COVID-19 pandemic is a complex global challenge, the timely actions conducted by the Vietnamese authorities are appreciated based on behavioral economics [7]. The world is accelerating to find a way to cope with the COVID-19 outbreaks; therefore, there are enormous studies in different fields to contribute to the COVID-19 containment, including the virology, public health, epidemic studies and others. At that time, these studies must trade-off thoroughness against speed, at least to some extent. Therefore, although this view is based on a single country, our perspectives are intended to prioritize the effective policies which are done by a developing country, Vietnam.
